# Associations between modifiable lifestyle behaviors and mental health indicators in adolescents from 48 countries: a cross-sectional study

**DOI:** 10.1186/s12887-025-06463-1

**Published:** 2026-01-05

**Authors:** Eduardo Rossato de Victo, Gerson Ferrari, Clemens Drenowatz, Dirceu Solé

**Affiliations:** 1 Division of Allergy and Clinical Immunology and Rheumatology, Department of Pediatrics, Universidade Federal de São Paulo - Escola Paulista de Medicina, São Paulo, Brazil; 2 Núcleo de Investigación en Ciencias del Movimiento, Universidad Arturo Prat, Iquique, Chile; 3 Escuela de Ciencias de la Actividad Física, el Deporte y la Salud, Universidad de Santiago de Chile (USACH), Santiago, Chile; 4 Facultad de Ciencias de la Salud, Universidad Autónoma de Chile, Providencia, Chile; 5 Division of Sport, Physical Activity and Health, University of Education Upper Austria, Linz, Austria

**Keywords:** Adolescent, Mental health, Suicide prevention, Anxiety, Loneliness, Lifestyle, Human development index, Multinational study

## Abstract

**Background:**

Lifestyle factors are known to be associated with mental health indicators. However, further studies are needed to explore how these associations manifest across different sociodemographic contexts. This study aimed to evaluate the association between modifiable lifestyle behaviors and mental health indicators among adolescents from 48 countries participating in the Global School-Based Student Health Survey (GSHS), considering each country’s Human Development Index (HDI) to account for sociodemographic differences.

**Methods:**

This cross-sectional study used data from national surveys conducted through the GSHS, including school-aged adolescents (10–19 years). Modifiable behaviors (obesity, fruit and vegetable consumption, physical activity, sitting time, transportation to school, tobacco and alcohol consumption and school attendance) and mental health indicators (anxiety, loneliness, suicidal thoughts and suicide planning) were obtained by self-reported questionnaire and categorized.

**Results:**

The final sample comprised 146,177 adolescents (54.2% female). Obesity, low fruit and vegetable consumption, physical inactivity, tobacco and alcohol use, and low school attendance were significantly associated with poor mental health indicators. Tobacco and alcohol use were strongly associated with anxiety (OR: 1.91; 95% CI: 1.81–2.02 and OR: 1.65; 95% CI: 1.58–1.73, respectively), loneliness (OR: 1.65; 95% CI: 1.57–1.74 and OR: 1.40; 95% CI: 1.34–1.46), suicidal ideation (OR: 2.57; 95% CI: 2.46–2.69 and OR: 2.06; 95% CI: 1.99–2.14), and suicide planning (OR: 2.49; 95% CI: 2.38–2.61 and OR: 1.98; 95% CI: 1.91–2.06), regardless of sex, age, and region. The strength of these associations varied according to country HDI.

**Conclusion:**

The associations between lifestyle behaviors and mental health indicators varied across HDI categories. However, tobacco use, alcohol consumption, and inadequate school attendance were consistently associated with anxiety, loneliness, suicidal ideation, and suicide planning at all HDI levels. These modifiable factors represent key targets for prevention strategies, underscoring the need for interventions that consider socioeconomic disparities.

**Supplementary Information:**

The online version contains supplementary material available at 10.1186/s12887-025-06463-1.

## Introduction

 In 2001, the World Health Organization (WHO) published its first report on mental health [1]. One of the ten written recommendations also emphasized the need for additional research on the biological and psychosocial aspects of mental health to enhance the understanding of mental disorders (or disorders) [1]. The report also reinforced the importance of research on a broad international scale and, mainly, with developing countries [1]. According to the WHO, mental health is defined as “a state of well-being and effective functioning in which an individual realizes his or her own abilities, is resilient to the stresses of life and is able to make a positive contribution to his or her community” [2].

It is known that physical and mental health influence each other through two main routes, physiological and behavioral [1]. Healthy behaviors play an important role in overall physical and mental health, while mental disorders can lead to risky behaviors. Anxiety, loneliness, depression and suicidal ideation are currently known mental disorders that are increasingly affecting people around the world, especially teenagers [3].

It is estimated that one in five adolescents experiences at least one mental health problem at this stage [4]. The first sign of these disorders generally occurs in childhood or adolescence, and half of all mental disorders in adulthood begin in adolescence [5, 6]. Today, the WHO recognizes suicide and depression as one of the main mental health problems among young people [7].

Despite the knowledge we have about the role of lifestyle and the association of healthy behaviors with the prevention of mental disorders as well as physical and mental health, studies are still needed to investigate predictive, moderating and mediating behaviors, especially among adolescents in developing countries with a low Human Development Index (HDI). The HDI is a widely recognized and comparable indicator that integrates dimensions of health, education, and income, providing a broader picture of human development than alternative measures such as gross domestic product or income inequality indices. Furthermore, the HDI allows us to contextualize results according to each country’s development. To improve care and public policies related to adolescent mental health, research is needed to identify and explore these associated factors [8].

Conceptually, the association between lifestyle behaviors and mental health is shaped by broader socioeconomic conditions [9, 10]. While lifestyle factors may influence psychological well-being through physiological and psychosocial pathways, the HDI reflects structural determinants (such as access to health resources, educational opportunities, and environmental stressors) that can modify these relationships [11]. Thus, the magnitude and direction of associations between lifestyle behaviors and mental health may differ across levels of socioeconomic development.

Evidence linking modifiable lifestyle behaviors to adolescent mental health is mixed. While physical activity is consistently associated with lower psychological distress [12], and substance use with a higher risk of mental health problems [13, 14], associations for other behaviors are less clear. For example, studies on obesity and dietary patterns have shown inconsistent results or associations that depend on the outcome assessed [15–17]. Similarly, the relationship between tobacco use and depression is complex and may be bidirectional [14]. Irregular school attendance has also been associated with symptoms of anxiety and depression [18]. These findings suggest that the effects of unhealthy behaviors on mental health may vary and require more comprehensive investigation.

Although several studies have documented the relationship between unhealthy lifestyle behaviors and mental health indicators in adolescents, most of these studies focus on regions and contexts with higher HDI, such as Europe and North America [12–19]. Data from lower-HDI regions and other geographic areas remain scarce, particularly regarding a broader assessment of lifestyle behaviors across multiple mental health indicators, such as anxiety, loneliness, suicidal ideation, and suicide planning. Furthermore, there is a gap in the literature regarding this relationship according to HDI, despite its relevance for understanding structural inequalities. By addressing these gaps, the present study provides comprehensive multinational evidence on the associations between modifiable behaviors and mental health among adolescents, with attention to the socioeconomic context.

Therefore, the objective of this study was to evaluate the association between modifiable lifestyle behaviors and mental health indicators among adolescents from 48 countries participating in the Global School-Based Student Health Survey (GSHS), with analyses stratified by each country’s HDI to account for socioeconomic differences.

## Methods

### Study design

For this cross-sectional study, data from national surveys obtained by the GSHS were used [20]. The data are publicly available, and details about the research can be found on the official WHO websites [21]; other publications have already detailed the project [20, 22, 23]. Briefly, the GSHS is a collaborative surveillance project whose objective is to help countries measure and evaluate behavioral and health factors in several key areas (eating behaviors, drug use, hygiene, mental health, physical activity, tobacco use, violence and unintentional injuries) among young people between 10 and 19 years of age.

The questionnaire was completed during regular school hours, digitized and translated into the corresponding language of the country. The questionnaire was validated, and content was extracted from the CDC Youth Risk Behavior Survey (YRBS), in which test and retest reliability has already been established [24].

### Ethical considerations

All procedures conducted in the GSHS complied with international standards for the protection of human subjects. The study received ethical approval in each participating country, typically through national ministries such as Health or Education and local ethics committees. The protocols followed guidelines established by the WHO. Participation in the survey was voluntary, with informed assent provided by the adolescents and consent obtained from their parents or legal guardians [25]. All procedures adhered to the ethical principles outlined in the Declaration of Helsinki.

### Sample

The research used a two-stage sampling technique to select schools and students for the study. At the initial stage, schools were selected with a probability proportional to the size of the student enrollment, and at the second stage, the classes were randomly selected, and all students aged 10 to 19 years in the selected class who met the eligibility criteria were recruited for inclusion in the study [20, 23, 26].

For this study, we used data collected and available on the GSHS website until June 2020. If there were more than two sets of data from the same country during this period, we chose the most recent set of data [22]. Countries that presented different data (rural or urban, public or private government, etc.) and national data and files with national data were considered.

A total of 388,381 adolescents from 102 countries initially composed the dataset. Participants without valid data for at least one of the study variables or without information about the country were excluded. As a result, 54 countries were entirely excluded because no eligible participants remained after applying these criteria. The final analytic sample therefore comprised 146,177 adolescents from 48 countries. For these countries, the initial available sample was 217,593 adolescents, of whom 71,416 were excluded due to missing data.

Finally, countries from the following regions participated: Africa (Benin, Botswana, Ghana, Liberia, Kenya, Mauritius, Mozambique, Namibia, Seychelles, Uganda, Tanzania); Europe (North Macedonia); Latin America and the Caribbean (Antigua and Barbuda, Argentina, Bahamas, Bolivia, Costa Rica, Ecuador, Grenada, Guyana, Honduras, Jamaica, Peru, Saint Lucia, Saint Vincent and the Grenadines, Suriname, Trinidad and Tobago, Uruguay); Eastern Mediterranean (Lebanon); Western Pacific (Brunei Darussalam, China, Fiji, Kiribati, Lao PDR, Malaysia, Mongolia, Philippines, Samoa, Solomon Islands, Tonga, Vanuatu); Southeast Asia (Bangladesh, Bhutan, Indonesia, Nepal, Sri Lanka, Thailand, East Timor). Country-specific attrition rates for the 48 retained countries are provided in Supplementary Table 1.

All variables in this study were obtained through a self-administered questionnaire. The questions, answer options and categorization of responses are described in Table 1.

**Table 1:** Independent and dependent variables of the study and their respective categorizations

Variables	Question	Answer options	Categorization
Sex	What is your gender?	Male or female	Male / Female
Age	How old are you?	≤ 11 years to ≥ 16 years	early adolescence (11–14 years) / late adolescence (≥ 15 years)
Height	How tall are you without shoes?	cm	-
Weight	How much do you weigh without shoes?	kg	-
Nutritional status	Calculated according to answers about height and weight (BMI).	-	Not obese/obese
Fruit consumption	During the last 30 days, how many times a day did you normally eat fruit?	1 = I have not eaten fruit in the last 30 days to 7 = ≥ 5 times/day	*≥* 2 = adequate consumption / <2 = inadequate consumption
Vegetable consumption	During the last 30 days, how many times a day did you usually eat vegetables?	1 = I have not eaten vegetables in the last 30 days to 7 = ≥ 5 times/day	*≥* 3 = adequate consumption / < 3 = inadequate consumption
Fruit and Vegetable Consumption	Calculated according to the answers to the questions about fruits and vegetables and classified according to the recommendations of > 2 fruits/day and *≥* 3 vegetables/day.	-	adequate consumption / inadequate consumption
Physical activity	During the last 7 days, how many days have you been physically active for a total of at least 60 min per day?	0 = 0 days to 7 = 7 days	0 day = inactive / / ≥1 days = practices PA
Sitting time	How much time do you spend during a typical or habitual day sitting and watching television, playing computer games, talking to friends, or doing other activities while sitting?	1 = Less than 1 h per day…3 = 3 to 4 h per day…6 = ≥ 8 h/day	Acceptable sitting time / excessive sitting time
Active transport	During the last 7 days, how many days did you walk or bike to or from school?	0 = 0 days to 7 = 7 days	Passive transport / active transport
Alcohol use	During the last 30 days, how many days have you had at least one drink containing alcohol?	1 = 0 days to 7 = All 30 days	No alcohol consumption / alcohol consumption
Tobacco use	During the last 30 days, how many days did you smoke cigarettes/use any tobacco products?	1 = 0 days to 7 = All 30 days	No tobacco use / tobacco use
School attendance	In the last 30 days, how many days have you missed classes or school without permission?	1 = 0 days to 5 = ≥ 10 days	adequate / inadequate
Anxiety	During the past 12 months, how often have you been so worried about something that you couldn’t sleep at night?	1 = never to 5 = always	Yes / No
Loneliness	During the past 12 months, how often have you felt lonely?	1 = never to 5 = always	Yes / No
Suicidal ideation	During the past 12 months, have you ever seriously considered attempting suicide?	Yes or no	Yes / No
Suicide planning	During the past 12 months, have you made a plan about how you would attempt suicide?	Yes or no	Yes / No

### Modifiable behaviors

The modifiable behavioral variables are nutritional status, fruit and vegetable consumption, physical activity (PA), sitting time (ST), transportation to school, tobacco and alcohol consumption and school attendance.

The nutritional status was not included in the questionnaire; however, with the answers obtained about height and weight, the body mass index (BMI) was calculated using the weight (kg)/height (m)^2^ formula, and subsequently, the participants were classified according to the criteria from the WHO, considering age and sex, by standard deviations (SD). Nutritional status was classified as non-obese (< 2 SD) or obese (*≥* 2 SD) [27].

Fruit and vegetable consumption was classified according to the WHO recommendation of ≥ 5 servings/day (*≥* 2 fruits/day and *≥* 3 vegetables/day) [28]. Specific examples of fruits and vegetables in the country were referenced in both questions [29].

PA was categorized as inactive (0 days/week) or practicing PA (*≥* 1 day/week) according to the number of days the participants practiced at least 60 min of PA [30]. PA was defined as any activity that increased the heart rate or left the student breathless for some time. PA included leisure sports, exercising with friends or walking to school [31].

Total sitting time (ST) was defined as excessive when the participant spent ≥ 3 h/day sitting [32]. Participants were instructed to exclude sitting time during school time and when doing homework [25].

The use of active transport was considered when the participant responded that they went to or returned from school actively (on foot or bicycle) on at least 3 days of the week, that is, most of the school days of the week [33].

Alcohol and/or tobacco use was considered when the student responded on at least one day in the last 30 days [34].

School attendance was considered adequate when the student had not missed school in the last 30 days [34].

### Mental health indicators

The feelings of anxiety and loneliness were dichotomized into yes (most of the time/always) and no (never, rarely, sometimes) [35]. Suicidal ideation and suicide planning were classified as yes or no according to what was stated in the questionnaire.

### Sociodemographic variables

Information about the countries and the geographic region to which they belonged was extracted according to the country’s own database available on the WHO website [21]. According to age, students were categorized into two groups: 10–14 years (early adolescence) and 15 years and older (late adolescence) [36]. This categorization also corresponded to the median age in our sample.

The HDI data were obtained from the United Nations Development Program (UNDP) and are available on the official website [37]. The HDI is classified as low (< 0.550), medium (0.550 to 0.699), high (0.700 to 0.799) or very high (> 0.800) [38]. The HDI year considered was the same in which the country’s assessment by the GSHS occurred or the report closest to the year of participation in the GSHS. For participating countries between 2006 and 2008, the 2008 UNDP report was considered. States/territories that are not members of the United Nations are not included in the UNDP reports and were therefore excluded from the study.

Analyses were stratified by the HDI, thus accounting for differences in socioeconomic development between countries. The HDI was selected because it provides a composite measure of health, education, and income, capturing broader aspects of human development than single indicators. Socioeconomic development may influence both lifestyle behaviors and mental health outcomes through access to resources, healthcare, education, and social support [39–41].

### Statistical analysis

Descriptive statistics are presented as frequencies and percentages. Logistic regression analysis was used to calculate the odds ratio and its 95% confidence interval (95% CI) between the independent and dependent variables. Univariate and multivariate regression analyses were performed. Multivariate logistic regression values were adjusted for sex, age and region. The existence of possible multicollinearity between the independent variables and the adjustment variables (gender, age and region) was verified by the variance inflation factor (VIF) values (acceptable: <10) and tolerance (> 0.8). The permitted level of significance was *p* < 0.001. All analyses were performed using IBM SPSS Statistics for Windows, version 22. (SPSS Inc., IBM Corp., Armonk, New York, NY, USA).

## Results

Table 2 presents the prevalence rates of anxiety (7.8%), loneliness (10.0%), suicidal ideation (12.4%), and suicide planning (14.4%). The sample consisted of 54.2% female adolescents, with 53.5% aged 15 or older. Among them, 7.3% were classified as obese, 85.6% did not report adequate fruit and vegetable consumption, 24.6% were considered inactive, and 36.2% reported excessive ST. Tobacco and alcohol consumption in the last 30 days was 10.0% and 19.1%, respectively, with 24.8% having inadequate school attendance.

**Table 2:** Demographic, behavioral, and mental health characteristics of adolescents participating in the study

Total = 146,177	Anxiety		Loneliness		Suicidal ideation		Suicide planning	
	Yes 11,387 (7.8)	No 134,790 (92.2)	Yes 14,645 (10.0)	No 131,532 (90.0)	Yes 18,139 (12.4)	No 128,038 (87.6)	Yes 16,730 (11.4)	No 129,447 (88.6)
Sex								
Male = 67,008 (45.8)	4266 (6.4)	62,742 (93.6)	5234 (7.8)	61,774 (92.2)	6573 (9.8)	60,435 (90.2)	6319 (9.4)	60,689 (90.6)
Female = 79,169 (54.2)	7121 (9.0)	72,048 (91.0)	9411 (11.9)	69,758 (88.1)	11,566 (14.6)	67,603 (85.4)	11,566 (14.6)	67,603 (85.4)
Age group								
Early adolescence = 67,954 (46.5)	4243 (6.2)	63,711 (93.8)	5758 (8.5)	62,196 (91.5)	7909 (11.6)	60,045 (88.4)	7081 (10.4)	60,873 (89.6)
Late adolescence = 78,223 (53.5)	7144 (9.1)	71,079 (90.9)	8887 (11.4)	69,336 (88.6)	10,230 (13.1)	67,993 (86.9)	9649 (12.3)	68,574 (87.7)
Region								
Africa = 15,903 (10.9)	1994 (12.5)	13,909 (87.5)	1980 (12.5)	13,923 (87.5)	2685 (16.9)	13,218 (83.1)	3070 (19.3)	12,833 (80.7)
Europe = 1311 (0.9)	67 (5.1)	1244 (94.9)	75 (5.7)	1236 (94.3)	96 (7.3)	1215 (92.7)	66 (5.0)	1245 (95.0)
Latin America and the Caribbean = 36,031 (24.5)	3070 (8.5)	32,961 (91.5)	3730 (10.4)	32,301 (89.6)	5946 (16.5)	30,085 (83.5)	5322 (14.8)	30,709 (85.2)
Eastern Mediterranean = 3887 (2.7)	560 (14.4)	3327 (85.6)	489 (12.6)	3398 (87.4)	473 (12.2)	3414 (87.8)	317 (8.2)	3570 (91.8)
Western Pacific = 56,939 (39.0)	3870 (6.8)	53,069 (93.2)	5627 (9.9)	51,312 (90.1)	6330 (11.1)	50,609 (88.9)	5080 (8.9)	51,859 (91.1)
Southeast Asia = 32,106 (22.0)	1826 (5.7)	30,280 (94.3)	2744 (8.5)	29,362 (91.5)	2609 (8.1)	29,497 (91.9)	2875 (9.0)	29,231 (91.0)
HDI								
Low = 6071 (4.2)	801 (13.2)	5270 (86.8)	676 (11.1)	5395 (88.9)	1023 (16.9)	5048 (83.1)	1063 (17.5)	5008 (82.5)
Medium = 45,244 (31.0)	3294 (7.3)	41,950 (92.7)	4399 (9.7)	40,845 (90.3)	5224 (11.5)	40,020 (88.5)	5882 (13.0)	39,362 (87.0)
High = 75,499 (51.6)	5723 (7.6)	69,776 (92.4)	7785 (10.3)	67,714 (89.7)	9084 (12.0)	66,415 (88.0)	7304 (9.7)	68,195 (90.3)
Very high = 19,363 (13.2)	1569 (8.1)	17,794 (91.9)	1785 (9.2)	17,578 (90.8)	2808 (14.5)	1655 (85.5)	2481 (12.8)	16,882 (87.2)
Nutritional status								
Obese = 10,633 (7.3)	911 (8.6)	9722 (91.4)	1155 (10.9)	9478 (89.1)	1350 (12.7)	9283 (87.3)	1221 (11.5)	9412 (88.5)
Not obese = 135,544 (92.7)	10,476 (7.7)	125,068 (92.3)	13,490 (10.0)	122,054 (90.0)	16,789 (12.4)	118,755 (87.6)	15,509 (11.4)	120,035 (88.6)
Fruit and vegetable consumption								
Inadequate consumption = 125,150 (85.6)	9689 (7.7)	115,461 (92.3)	12,650 (10.1)	112,500 (89.9)	15,711 (12.6)	109,439 (87.4)	14,424 (11.5)	110,726 (88.5)
Adequate consumption = 21,027 (14.4)	1698 (8.1)	19,329 (91.9)	1995 (9.5)	19,032 (90.5)	2428 (11.5)	18,599 (88.5)	2306 (11.0)	18,721 (89.0)
Physical activity								
Inactive = 35,900 (24.6)	2938 (8.2)	32,962 (91.8)	3909 (10.9)	31,991 (89.1)	4791 (13.3)	31,109 (86.7)	4543 (13.3)	31,109 (86.7)
Practices physical activity = 110,277 (75.4)	8449 (7.7)	101,828 (92.3)	10,736 (9.7)	99,541 (90.3)	13,348 (12.1)	96,929 (87.9)	13,348 (12.1)	96,929 (87.9)
Sitting time								
Excessive sitting time = 52,946 (36.2)	4918 (9.3)	48,028 (90.7)	6371 (12.0)	46,575 (88.0)	7932 (15.0)	45,014 (85.0)	7000 (13.2)	45,946 (86.8)
Acceptable sitting time = 93,231 (63.8)	6469 (6.9)	86,762 (93.1)	8274 (8.9)	84,957 (91.1)	10,207 (10.9)	83,024 (89.1)	9730 (10.4)	128,038 (87.6)
Transport to school								
Passive = 84,592 (57.9)	6595 (7.8)	77,997 (92.2)	8347 (9.9)	76,425 (90.1)	10,218 (12.1)	74,374 (87.9)	9733 (11.5)	74,859 (88.5)
Active = 61,585 (42.1)	4792 (7.8)	56,793 (92.2)	6298 (10.2)	55,287 (89.8)	7921 (12.9)	53,664 (87.1)	6997 (11.4)	54,588 (88.6)
Tobacco consumption								
Yes = 14,572 (10.0)	1796 (12.3)	12,776 (87.7)	2015 (13.8)	12,557 (86.2)	3192 (21.9)	11,380 (78.1)	2959 (20.3)	11,613 (79.7)
No = 131,605 (90.0)	9591 (7.3)	122,014 (92.7)	12,630 (9.6)	118,975 (90.4)	14,947 (11.4)	116,658 (88.6)	13,771 (10.5)	117,834 (89.5)
Alcohol consumption								
Yes = 27,904 (19.1)	3377 (12.1)	24,527 (87.9)	3653 (13.1)	24,251 (86.9)	5778 (20.7)	22,126 (79.3)	5379 (19.3)	22,525 (80.7)
No = 118,273 (80.9)	8010 (7.8)	110,263 (93.2)	10,992 (9.3)	107,281 (90.7)	12,361 (10.5)	105,912 (89.5)	11,351 (9.6)	106,922 (90.4)
School attendance								
Inadequate = 36,386 (24.9)	3631 (10.0)	32,755 (90.0)	4484 (12.3)	31,902 (87.7)	5771 (15.9)	30,615 (84.1)	5515 (15.2)	30,871 (89.8)
Adequate = 109,791 (75.1)	7756 (7.1)	102,035 (92.9)	10,161 (9.3)	99,630 (90.7)	12,368 (11.3)	97,423 (88.7)	11,215 (10.2)	98,576 (89.8)

Prevalence estimates for all lifestyle behaviors stratified by HDI category are presented in Supplementary Table 2.

Table 3 illustrates the association between modifiable behavior variables and mental health indicators. Adjusted for sex, age, and region, being obese was positively associated with anxiety and loneliness compared to non-obese individuals. Inactivity, excessive sitting time, tobacco and alcohol consumption, and inadequate school attendance were positively associated with all mental health indicators compared to adolescents engaging in physical activity, those with appropriate sitting time, non-smokers and non-drinkers, and those with adequate school attendance. Inadequate fruit and vegetable consumption and passive transportation to school showed a negative association with anxiety and suicidal ideation compared to those with adequate fruit and vegetable consumption and active transportation to school.

**Table 3:** Modifiable factors of adolescents’ lifestyle and mental health – Adjusted logistic regression analysis

Modifiable behaviors	Anxiety		Loneliness		Suicidal ideation		Suicide planning	
	OR^1^ (CI95%)	OR^2^ (CI95%)	OR^1^ (CI95%)	OR^2^ (CI95%)	OR^1^ (CI95%)	OR^2^ (CI95%)	OR^1^ (CI95%)	OR^2^ (CI95%)
Nutritional status (ref.= Not obese)								
Obese	1.12 (1.04–1.20)**	1.21 (1.13–1.30)*	1.10 (1.03–1.17)**	1.19 (1.11–1.27)*	1.03 (0.97–1.09)	1.07 (0.01–1.14)**	1.00 (0.94–1.07)	1.05 (0.99–1.12)
Fruit and vegetable consumption (ref.= Adequate)								
Inadequate	0.95 (0.90–1.01)	0.90 (0.85–0.95)*	1.07 (1.02–1.13)**	1.03 (0.98–1.05)	1.10 (1.05–1.15)*	1.06 (1.01–1.10)**	1.06 (1.01–1.10)**	1.01 (0.96–1.05)
Physical activity (ref.= Practices PA)								
Inactive	1.07 (1.03–1.12)**	1.08 (1.04–1.13)*	1.13 (1.09–1.18)*	1.12 (1.08–1.17)*	1.12 (1.08–1.16)*	1.11 (1.07–1.15)*	1.17 (1.12–1.21)*	1.17 (1.13–1.21)*
Sitting time (ref.= Acceptable sitting time)								
Excessive time sitting	1.37 (1.32–1.43)*	1.29 (1.34–1.39)*	1.40 (1.36–1.35)*	1.34 (1.30–1.39)*	1.43 (1.39–1.48)*	1.37 (1.33–1.42)*	1.31 (1.26–1.36)*	1.25 (1.20–1.29)*
Transport to school (ref.= Active)								
Passive	1.00 (0.96–1.04)	1.02 (0.98–1.06)	0.96 (0.93–0.99)**	0.96 (0.93–0.99)**	0.93 (0.90–0.96)*	0.94 (0.91–0.97)*	1.01 (0.98–1.05)	1.03 (0.99–1.07)
Tobacco consumption (ref.= No)								
Yes	1.79 (1.69–1.89)*	1.91 (1.81–2.02)*	1.51 (1.44–1.39)*	1.65 (1.57–1.74)*	2.19 (2.10–2.29)*	2.57 (2.46–2.69)*	2.18 (2.09–2.28)*	2.49 (2.38–2.61)*
Alcohol consumption (ref.= No)								
Yes	1.89 (1.82–1.98)*	1.65 (1.58–1.73)*	1.47 (1.41–1.53)*	1.40 (1.34–1.46)*	2.24 (2.16–2.32)*	2.06 (1.99–2.14)*	2.25 (2.17–2.33)*	1.98 (1.91–2.06)*
School attendance (ref.= Adequate)								
Inadequate	1.46 (1.40–1.52)*	1.42 (1.37–1.49)*	1.38 (1.33–1.43)*	1.37 (1.32–1.42)*	1.48 (1.44–1.54)*	1.51 (1.46–1.57)*	1.57 (1.52–1.63)*	1.58 (1.53–1.64)*

1: univariate logistic regression, 2: multivariate logistic regression adjusted for sex, age and region

*p < 0.001; ** *p* < 0.05

Table 4 presents the results of the association between lifestyle factors and mental health indicators according to the HDI, adjusted for sex, age, and region. Being obese was associated with higher odds of suicidal ideation in the medium HDI group (OR: 1.31, 95% CI: 1.13–1.51) and anxiety and loneliness in the high HDI group (OR: 1.21, 95% CI: 1.10–1.32; OR: 1.16, 95% CI: 1.07–1.26, respectively). Inadequate fruit and vegetable consumption was positively associated with suicidal ideation in the high HDI group (OR: 1.14, 95% CI: 1.07–1.21). Being inactive was positively associated with anxiety (OR: 1.22, 95% CI: 1.15–1.30), suicidal ideation (OR: 1.18, 95% CI: 1.12–1.24), and suicide planning (OR: 1.18, 95% CI: 1.12–1.25) in the high HDI group. Excessive sitting time was positively associated only with suicidal ideation in the low HDI group, but in other HDI groups, it was positively associated with all mental health indicators. Passive transportation to school was negatively associated with anxiety in the low HDI group (OR: 0.45, 95% CI: 0.65–1.87), suicidal ideation in the high HDI group (OR: 0.878, 95% CI: 0.83–0.91), loneliness and suicide planning in the very high HDI group (OR: 0.80, 95% CI: 0.72–0.89; OR: 0.80, 95% CI: 0.74–0.88, respectively).

**Table 4:** Adjusted associations between lifestyle behaviors and mental health outcomes, stratified by HDI category

	HDI low				HDI medium			
Modifiable behaviors	Anxiety	Loneliness	Suicidal ideation	Suicide planning	Anxiety	Loneliness	Suicidal ideation	Suicide planning
Nutritional status (ref.= Not obese)								
Obese	1.11 (0.72–1.73)	0.81 (0.48–1.37)	1.14 (0.78–1.67)	1.12 (0.76–1.64)	1.17 (0.97–1.41)	1.15 (0.98–1.36)	1.31 (1.13–1.51)*	1.11 (0.96–1.29)
Fruit and vegetable consumption (ref.= Adequate)								
Inadequate	0.92 (0.73–1.15)	1.00 (0.78–1.27)	1.02 (0.83–1.25)	0.94 (0.77–1.14)	0.94 (0.84–1.04)	1.02 (0.93–1.12)	0.95 (0.87–1.03)	0.98 (0.90–1.06)
Physical activity (ref.= Practices PA)								
Inactive	0.91 (0.75–1.11)	1.01 (0.83–1.25)	1.02 (0.85–1.21)	1.10 (0.9–1.30)	0.91 (0.84–0.98)**	0.91 (0.85–1.97)	1.03 (0.97–1.09)	1.09 (1.03–1.16)**
Sitting time (ref.= Acceptable sitting time)								
Excessive time sitting	1.14 (0.96–1.35)	1.30 (1.01–1.55)**	1.38 (1.19–1.61)*	1.17 (1.01–1.36)**	1.42 (1.31–1.53)*	1.44 (1.34–1.54)*	1.38 (1.30–1.48)*	1.33 (1.25–1.41)*
Transport to school (ref.= Active)								
Passive	0.75 (0.64–0.87)*	0.81 (0.70–0.95)**	1.02 (0.89–1.17)	1.03 (0.90–1.18)	1.04 (0.97–1.12)	1.01 (0.94–1.07)	1.07 (1.01–1.14)**	1.10 (1.04–1.17)**
Tobacco consumption (ref.= No)								
Yes	2.00 (1.48–2.70)*	2.03 (1.49–2.77)*	2.60 (2.01–3.35)*	2.15 (1.66–2.80)*	2.00 (1.80–2.21)*	1.97 (1.79–2.16)*	2.77 (2.54–3.01)*	2.56 (2.36–2.78)*
Alcohol consumption (ref.= No)								
Yes	1.76 (1.49–2.07)*	1.48 (1.24–1.78)*	1.53 (1.31–1.79)*	1.39 (1.19–1.62)*	1.76 (1.62–1.92)*	1.57 (1.45–1.70)*	2.07 (1.93–2.23)*	1.91 (1.78–2.05)*
School attendance (ref.= Adequate)								
Inadequate	1.37 (1.17–1.60)*	1.38 (1.16–1.64)*	1.44 (1.25–1.67)*	1.54 (1.34–1.77)*	1.53 (1.42–1.65)*	1.29 (1.21–1.39)*	1.59 (1.50–1.70)*	1.61 (1.51–1.70)*
Modifiable behaviors	HDI high				HDI very high			
	Anxiety	Loneliness	Suicidal ideation	Suicide planning	Anxiety	Loneliness	Suicidal ideation	Suicide planning
Obese	1.21 (1.10–1.32)*	1.16 (1.07–1.26)*	1.01 (0.94–1.09)	1.12 (1.04–1.23)**	1.23 (1.03–1.48)**	1.29 (1.09–1.53)**	1.12 (0.96–1.31)	1.12 (0.96–1.32)
Fruit and vegetable consumption (ref.= Adequate)								
Inadequate	0.89 (0.83–0.95)**	1.05 (0.98–1.12)	1.14 (1.07–1.21)*	1.01 (0.95–1.08)	0.88 (0.74–1.04)	1.12 (0.94–1.33)	1.10 (0.95–1.26)	1.14 (0.98–1.32)
Physical activity (ref.= Practices PA)								
Inactive	1.22 (1.15–1.30)*	1.28 (1.21–1.35)*	1.18 (1.12–1.24)*	1.18 (1.12–1.25)*	1.14 (0.98–1.31)	1.12 (0.98–1.28)	1.16 (1.04–1.30)**	1.12 (0.99–1.26)
Sitting time (ref.= Acceptable sitting time)								
Excessive time sitting	1.27 (1.30–1.34)*	1.32 (1.26–1.38)*	1.39 (1.33–1.45)*	1.36 (1.29–1.42)*	1.32 (1.19–1.47)*	1.31 (1.19–1.45)*	1.33 (1.23–1.44)*	1.35 (1.23–1.47)*
Transport to school (ref.= Active)								
Passive	1.08 (1.02–1.14)**	0.97 (0.92–1.02)	0.87 (0.83–0.91)*	1.03 (0.98–1.08)	0.86 (0.77–0.95)**	0.80 (0.72–0.89)*	0.93 (0.86–1.02)	0.80 (0.74–0.88)*
Tobacco consumption (ref.= No)								
Yes	2.04 (1.87–2.21)*	1.60 (1.48–1.73)*	2.31 (2.16–2.48)*	2.37 (2.20–2.55)*	1.91 (1.68–2.17)*	1.58 (1.40–2.79)*	2.83 (2.57–3.11)*	2.78 (2.51–3.07)*
Alcohol consumption (ref.= No)								
Yes	1.88 (1.76-2.00)*	1.56 (1.47–1.66)*	2.27 (2.15–2.39)*	2.33 (2.20–2.47)*	1.61 (1.44–1.80)*	1.26 (1.14–1.41)*	2.12 (1.94–2.31)*	2.34 (2.13–2.57)*
School attendance (ref.= Adequate)								
Inadequate	1.40 (1.31–1.48)*	1.37 (1.30–1.45)*	1.42 (1.35–1.49)*	1.49 (1.41–1.58)*	1.33 (1.19–1.49)*	1.53 (1.38–1.70)*	1.62 (1.49–1.77)*	1.62 (1.48–1.77)*

HDI: Human Development Index

Analyses were performed separately for each HDI category; formal statistical tests for interaction were not conducted

Multivariate regression values adjusted by sex, age and region

*p < 0.001; ** *p* < 0.05

Alcohol and tobacco consumption were positively associated with all mental health indicators in all HDI groups. The same pattern was observed for the factor of inadequate school attendance (Table 4).

## Discussion

The present study aimed to evaluate the association between modifiable lifestyle behaviors and mental health indicators among adolescents from 48 countries participating in the GSHS, considering each country’s HDI to account for sociodemographic differences. The analyses by HDI, clear heterogeneity emerged across socioeconomic contexts. In low-HDI countries, excessive sitting time showed a positive association with suicidal ideation, whereas passive transportation to school exhibited a negative association with anxiety. In medium-HDI settings, obesity was positively associated with suicidal ideation, and excessive sitting time demonstrated positive associations with anxiety, loneliness, suicidal ideation, and suicide planning. In high-HDI countries, several associations were stronger and consistently positive, including those between obesity and anxiety/loneliness, inadequate fruit and vegetable consumption and suicidal ideation, and both physical inactivity and excessive sitting time with all mental health indicators. In this group, passive transportation showed a negative association with suicidal ideation. In very-high-HDI countries, excessive sitting time was again positively associated with anxiety, loneliness, suicidal ideation, and suicide planning, while passive transportation displayed negative associations with loneliness and suicide planning. Across all HDI levels, tobacco use, alcohol consumption, and inadequate school attendance showed positive associations with anxiety, loneliness, suicidal ideation, and suicide planning, suggesting that these behaviors confer similar risks irrespective of socioeconomic development.

Regarding the prevalence of mental health indicators, our data showed that at least one in ten adolescents presented mental health indicators. Among the regions participating in this study, Africa and the Eastern Mediterranean had the highest prevalence of anxiety and loneliness, while suicidal ideation and suicide planning were more common in the American and African regions. The observed prevalence is consistent with the prevalence previously estimated by other studies [42, 43]. In an annual research review using a meta-analysis of 41 studies and 27 countries, the prevalence of mental health problems in children and adolescents was 13.4% [42]. Similarly, a report on mental health indicated a prevalence ranging between 10% and 20% in children and adolescents around the world who had a mental health problem [43]. According to these authors, although only 10% of the studies were in low- and middle-income countries, approximately 90% of children around the world live in countries with these conditions, suggesting the need for more studies in these regions [43]. It is important to highlight that the present study shows data that, for the most part, are from countries that are not part of the world’s large economies, including data from low- and medium-HDI countries. This is important, as it contributes evidence from regions that remain underrepresented in global mental health research, helping fill important knowledge gaps in highly populated areas with fewer available studies [43].

The present study showed that 7% of the adolescents in the sample were classified as obese, and these individuals had greater chances of presenting anxiety and loneliness compared with their non-obese peers. For this study, we chose to classify nutritional status into obese and non-obese people to investigate the associations of extreme nutritional status, in addition to adjusting for sex and age. These data corroborate the findings of a previous study that showed a linear association between BMI and psychological well-being [44]. A study with adolescents from Latin America and the Caribbean investigated the association between nutritional status and suicide, respecting the economic particularities of each country, and showed that suicidal ideation and planning were not associated with the country’s level of development but rather with overweight and obesity [45]. In the present study, obesity was associated with mental health indicators in general, but when analyzed by HDI, the values were significant only in the medium- and high-HDI groups.

A systematic review of data from Chinese adolescents showed that the prevalence of depression and anxiety among those who were overweight or obese was greater than that among those who were not overweight or obese [46]. To clarify these findings, we consider that children and adolescents who are overweight or obese tend to suffer more frequently from social problems, such as bullying and social marginalization [47, 48]. The data also showed that these mental health and psychological well-being problems in adolescence may be more significant during the first years of adolescence, but in the final years of adolescence, the impact may be less, perhaps justified by the different reactions and sensitivities that the adolescent has at each stage of this sensitive period [44]. Our data, however, confirmed a positive association between mental health problems (anxiety and loneliness) and obesity even after the analyses were adjusted.

Inadequate consumption of fruits and vegetables showed inconsistent associations in our study, being linked to lower odds of anxiety but higher odds of suicidal ideation. The literature supports the importance of a healthy diet for both physical and mental health, emphasizing a diet with a good variety and sufficient quantity of fruits and vegetables, and limited pro-inflammatory foods such as fast food and junk food [49–51]. Inadequate fruit and vegetable intake may also be associated with other health consequences, such as overweight and obesity, which could partly explain the higher likelihood of poorer mental health outcomes. Additionally, nutrients present in fruits and vegetables may play a protective role in mental health [52]. However, because our study assessed only adequate versus inadequate fruit and vegetable consumption, the measure captures a very limited aspect of overall diet quality. This prevents a more detailed exploration of dietary patterns. Furthermore, low intake does not necessarily reflect an unhealthy diet, as individuals with low intake may still have balanced eating habits, while others with adequate intake may engage in unhealthy dietary behaviors. As a result, this single-item measure may generate inconsistent or counterintuitive associations, potentially influenced by residual confounding and differences in dietary habits across countries.

The present study only measured the frequency of PAs and did not consider the intensity with which these activities were performed. Additionally, the answers were limited to knowing whether PA practice occurred for at least 60 min/day, not allowing an answer about the total accumulated time of PA during the day or during the week, which led to a greater probability of error in the classification of the AF level. This limitation of the study is that we preferred to classify the sample as those who practice PA rather than those who are inactive to analyze the effect of physical inactivity on mental health. Our results suggest that inactivity is associated with higher odds of worse mental health compared to those who performed some PAs during the week. Other studies also reported the health benefits of practicing at least one or two PA sessions a week [53, 54]. According to the findings, not being inactive can represent an important mental health behavior.

Participants with excessive ST were more likely to have worse mental health in the present study, as has been shown previously [55]. Even so, for now, little is known about the interrelationships that explain this phenomenon [56]. We can speculate that excessive ST favors moments of “bad” thoughts due to excessive idleness compared to those who exchange their ST for other activities that involve movement, for example, PA. Furthermore, excess ST may be combined with other habits that are harmful to health, such as physical inactivity and consequently obesity [57]. As we do not have more specific and detailed data on ST, these hypotheses are limited to the field of speculation.

Contrary to our initial hypothesis, considering active transportation as a healthy behavior linked to physical activity, passive transportation to school showed a negative association with the outcome of suicidal ideation. Perhaps, a portion of adolescents using passive transportation to school are driven by their parents or relatives, and this could positively contribute to feelings of loneliness and lower chances of suicidal ideation. Another speculation revolves around environmental factors that may influence the sensations experienced by adolescents based on the mode of transportation used, taking into account the barriers faced by those using active transportation, such as safety concerns, the burden of carrying a backpack, routes, and social aspects [58].

Alcohol and tobacco consumption were significantly associated with higher odds of worse mental health indicators. These behaviors are a major challenge for public health and are generally associated with mental health indicators [59]. Our data corroborate existing data on the negative associations of alcohol and tobacco use with various indicators of mental health [60–62]. Studies have shown that alcohol and tobacco are risk factors for adolescents’ health and are associated with anxiety, depression and suicidal behavior [61, 63]. In fact, tobacco use is an independent risk factor for poor mental health [64]. Alcohol and tobacco are important factors related to suicide, and more studies are needed to investigate their harmful effects on the brain and, consequently, on mental health [60, 62]. Although there are data that show better psychological well-being with a greater frequency of drinking, these data are not consistent, and it is very likely that the results are biased by confounding factors [62].

Inadequate school attendance was associated with worse mental health indicators in the present study, consistent with findings from previous research [65]. Systematic reviews have shown associations of inadequate school attendance with anxiety disorders, depression and social phobia, and researchers warn that school attendance should also be considered by doctors and school staff, as it can serve to enhance mental health [66, 67]. However, it is important to note that these associations may be bidirectional. Additionally, school attendance was assessed over only a one-month period in the present study, which limits conclusions about longer-term effects.

The present study also analyzed the associations between lifestyle factors and mental health indicators according to the HDI for better external validity of the results and generalization of the data, as this is a study with a large number of countries and differences in cultural, economic and social significance. Associations with some factors and indicators differed according to the HDI. Studies that analyzed outcomes affected by the HDI, such as obesity and PA, also reported different results according to the HDI [41, 68]. However, tobacco and alcohol consumption and school attendance were associated with all HDI classifications and all mental health indicators. Therefore, we believe that these results indicate that such behaviors seem to have the greatest impact on mental health among the behaviors analyzed here. The fact that they were the most significant values in all mental health indicators and in all HDI classifications reinforces our interpretation.

Overall, these findings suggest that while some associations vary depending on socioeconomic context, others are observed across contexts. The association between obesity and anxiety, especially in countries with higher HDI, may reflect contextual factors such as stronger social stigma related to body image [69]. Our hypothesis is that in more socioeconomically developed countries, adolescents may face more intense pressures regarding body standards imposed by media, cultural norms, and peer expectations, which may contribute to higher levels of anxiety. In contrast, alcohol and tobacco use may influence mental health primarily through biological rather than social mechanisms. This may partly explain why the use of these substances was consistently associated with worse mental health outcomes regardless of HDI. These findings highlight the importance of stratifying analyses by socioeconomic determinants such as HDI, since some associations between lifestyle behaviors and mental health appear to depend on context, whereas others remain consistent across HDI levels, allowing for a more refined understanding of how socioeconomic development interacts with adolescent health behaviors.

It is important to recognize that modifiable lifestyle behaviors frequently co-occur and may exert combined, rather than independent, effects [70, 71]. Although our study reports associations between individual behaviors and mental health indicators, these behaviors can interact in complex ways. Consequently, the observed associations should not be interpreted as causal, and the associations for individual behaviors represent only a partial view of the broader interplay among lifestyle factors.

The present study has other limitations, in addition to those mentioned above: (1) the cross-sectional nature of the study does not allow the establishment of causalities between the variables; (2) the use of questionnaires, even if validated, is subject to incorrect responses from adolescents, recall bias and other errors that could reduce the power of associations; (3) the generalization of data should be viewed with caution, as there was significant sample loss in many countries due to incomplete responses or data; (4) the data were collected across a long time span (2003–2017), and socioeconomic conditions of the participating countries may have changed since then, which may limit the contemporary applicability of the findings; and (5) multilevel analyses could not be performed because the dataset lacked the necessary information to identify and structure the school-level hierarchy. Despite this limitation, all analyses were stratified by the countries’ HDI, which allowed us to partially account for relevant contextual differences. Even so, this is a study with a significant sample of school-age adolescents from 48 countries around the world and six major global areas, in addition to involving heterogeneous economic levels and cultural aspects. The data were obtained through a rigorous standard of training, evaluation and collection, and the results have been published in the main journals in the field [23, 25, 33, 45].

The present study contributes to the WHO recommendation in the search for a better understanding of the factors related to mental disorders in adolescents by presenting data from countries outside the world’s main economies and with different HDIs. By complementing findings from other international, school-based health surveys conducted predominantly in high-income countries [19, 56, 72, 73], our study broadens the current evidence base by including adolescents from a wider spectrum of socioeconomic and cultural contexts, as well as other global regions, particularly in low- and middle-income countries. This contribution strengthens the global understanding of how lifestyle behaviors relate to adolescent mental health across diverse settings [19]. Furthermore, this study raises questions about possible factors that should be considered in future studies on adolescent mental health, such as school attendance. The consumption of alcohol and tobacco is also essential for future analyses. Identifying and exploring factors related to mental health may help guide care and public policies in countries in the search for prevention and care for the mental health of young people around the world. Longitudinal studies are needed to clarify the mechanisms of associated factors, and future research using approaches such as mixed-effects models could better capture the combined effects of lifestyle behaviors on adolescent mental health.

## Conclusion

The associations between lifestyle behaviors and mental health indicators varied across HDI categories. However, tobacco use, alcohol consumption, and inadequate school attendance were consistently associated with anxiety, loneliness, suicidal ideation, and suicide planning at all HDI levels, suggesting that these behaviors confer similar risks regardless of socioeconomic development.

As these risk factors are modifiable, they represent important targets for prevention strategies. Public health policies aiming to improve adolescent mental health should prioritize interventions addressing these behaviors while considering socioeconomic disparities reflected by the HDI.

## Supplementary Information

Supplementary Material 1.

## Data Availability

This document uses data from the Global School-Based Student Health Survey (GSHS). The GSHS is maintained by the World Health Organization and the United States Centers for Disease Control and Prevention. All data from this study can be found on the website:https://www.who.int/teams/noncommunicable-diseases/surveillance/systems-tools/global-school-based-student-health-survey.
