# Supplementary material for: Associations between modifiable lifestyle behaviors and mental health indicators in adolescents from 48 countries: a cross-sectional study
Source: BMC Pediatr. 2026 Jan 5;26:103. doi: 10.1186/s12887-025-06463-1 (PMC12896156; doi:10.1186/s12887-025-06463-1)
Supplement: Supplementary file 1 — Supplementary Material 1. [file 12887_2025_6463_MOESM1_ESM.docx]

| Supplementary Table 1: Sample distribution and country-specific attrition rates. | | | | | | | | |
| --- | --- | --- | --- | --- | --- | --- | --- | --- |
| Country | Region | Year | HDI category | Initial sample | Excluded | Final sample | Percentage of final sample | Attrition Rate (%) |
| Antigua and Barbuda | America | 2009 | High | 1266 | 1105 | 161 | 0.1 | 87.3 |
| Argentina | America | 2012 | Very high | 28368 | 14405 | 13963 | 9.6 | 50.8 |
| Bahamas | America | 2013 | High | 1357 | 362 | 995 | 0.7 | 26.7 |
| Bangladesh | Southeast Asia | 2014 | Medium | 2989 | 599 | 2390 | 1.6 | 20.0 |
| Benin | African | 2016 | Low | 2536 | 436 | 2100 | 1.4 | 17.2 |
| Bhutan | Southeast Asia | 2016 | Medium | 7576 | 1071 | 6505 | 4.5 | 14.1 |
| Bolivia | America | 2012 | Medium | 3696 | 819 | 2877 | 2.0 | 22.2 |
| Botswana | African | 2005 | Medium | 2197 | 2089 | 108 | 0.1 | 95.1 |
| Brunei Darussalam | Western Pacific | 2014 | Very high | 2599 | 252 | 2347 | 1.6 | 9.7 |
| China | Western Pacific | 2003 | High | 9015 | 2355 | 6660 | 4.6 | 26.1 |
| Costa Rica | America | 2009 | High | 2679 | 366 | 2313 | 1.6 | 13.7 |
| East Timor | Southeast Asia | 2015 | Medium | 3704 | 1397 | 2307 | 1.6 | 37.7 |
| Ecuador | America | 2007 | Very high | 5524 | 3844 | 1680 | 1.1 | 69.6 |
| Fiji | Western Pacific | 2016 | High | 3705 | 847 | 2858 | 2.0 | 22.9 |
| Ghana | African | 2012 | Medium | 3632 | 711 | 2921 | 2.0 | 19.6 |
| Grenada | America | 2008 | High | 1542 | 1513 | 29 | 0.0 | 98.1 |
| Guyana | America | 2010 | Medium | 2392 | 567 | 1825 | 1.2 | 23.7 |
| Honduras | America | 2012 | Medium | 1779 | 361 | 1418 | 1.0 | 20.3 |
| Indonesia | Southeast Asia | 2015 | Medium | 11142 | 1475 | 9667 | 6.6 | 13.2 |
| Jamaica | America | 2017 | High | 1667 | 390 | 1277 | 0.9 | 23.4 |
| Kenya | African | 2003 | Low | 3691 | 3430 | 261 | 0.2 | 92.9 |
| Kiribati | Western Pacific | 2011 | Medium | 1582 | 410 | 1172 | 0.8 | 25.9 |
| Lao People Democratic Republic | Western Pacific | 2015 | Medium | 3683 | 386 | 3297 | 2.3 | 10.5 |
| Lebanon | Eastern Mediterranean | 2017 | High | 5708 | 1821 | 3887 | 2.7 | 31.9 |
| Liberia | African | 2017 | Low | 2744 | 2723 | 21 | 0.0 | 99.2 |
| Malaysia | Western Pacific | 2012 | High | 25507 | 2057 | 23450 | 16.0 | 8.1 |
| Mauritius | African | 2017 | High | 3012 | 539 | 2473 | 1.7 | 17.9 |
| Mongolia | Western Pacific | 2013 | High | 5393 | 535 | 4858 | 3.3 | 9.9 |
| Mozambique | African | 2015 | Low | 1918 | 1534 | 384 | 0.3 | 80.0 |
| Namibia | African | 2013 | Medium | 4531 | 1015 | 3516 | 2.4 | 22.4 |
| Nepal | Southeast Asia | 2015 | Medium | 6529 | 1491 | 5038 | 3.4 | 22.8 |
| North Macedonia | European | 2007 | Very high | 2114 | 803 | 1311 | 0.9 | 38.0 |
| Peru | America | 2010 | High | 2882 | 641 | 2241 | 1.5 | 22.2 |
| Philippines | Western Pacific | 2015 | High | 8761 | 2148 | 6613 | 4.5 | 24.5 |
| Saint Lucia | America | 2007 | Very high | 1276 | 1214 | 62 | 0.0 | 95.1 |
| Saint Vicent and the Grenadines | America | 2007 | High | 1333 | 1296 | 37 | 0.0 | 97.2 |
| Samoa | Western Pacific | 2011 | Medium | 2418 | 1568 | 850 | 0.6 | 64.8 |
| Seychelles | African | 2015 | High | 2540 | 880 | 1660 | 1.1 | 34.6 |
| Solomon Islands | Western Pacific | 2011 | Low | 1421 | 575 | 846 | 0.6 | 40.5 |
| Sri Lanka | Southeast Asia | 2016 | High | 3262 | 1726 | 1536 | 1.1 | 52.9 |
| Suriname | America | 2016 | High | 2126 | 413 | 1713 | 1.2 | 19.4 |
| Thailand | Southeast Asia | 2015 | High | 5894 | 1231 | 4663 | 3.2 | 20.9 |
| Tonga | Western Pacific | 2017 | High | 3333 | 698 | 2635 | 1.8 | 20.9 |
| Trinidad and Tobago | America | 2017 | High | 3869 | 1099 | 2770 | 1.9 | 28.4 |
| Uganda | African | 2003 | Low | 3215 | 1012 | 2203 | 1.5 | 31.5 |
| UR Tanzania | African | 2014 | Low | 3793 | 3537 | 256 | 0.2 | 93.3 |
| Uruguay | America | 2012 | High | 3524 | 854 | 2670 | 1.8 | 24.2 |
| Vanuatu | Western Pacific | 2016 | Medium | 2159 | 806 | 1353 | 0.9 | 37.3 |
| From the initial 102 countries (n = 388,381), 48 countries remained after exclusions (initial available n = 217,593), yielding a final analytic sample of 146,177 participants. | | | | | | | | |

| Supplementary Table 2: Prevalence of lifestyle risk behaviors according to the HDI. | | | | |
| --- | --- | --- | --- | --- |
| Risk behaviors | HDI low | HDI medium | HDI high | HDI very high |
|  | % | N (%) | N (%) | N (%) |
| Nutritional status (ref.= Not obese) |  |  |  |  |
| Obese | 3.1 | 3.7 | 9.4 | 8.5 |
| Fruit and vegetable consumption (ref.= Adequate) |  |  |  |  |
| Inadequate | 87.3 | 86.3 | 84.0 | 85.6 |
| Physical activity (ref.= Practices PA) |  |  |  |  |
| Inactive | 19.0 | 31.5 | 23.6 | 13.9 |
| Sitting time (ref.= Acceptable sitting time) |  |  |  |  |
| Excessive time sitting | 24.8 | 23.1 | 42.0 | 48.1 |
| Transport to school (ref.= Active) |  |  |  |  |
| Passive | 51.1 | 62.9 | 57.0 | 51.5 |
| Tobacco consumption (ref.= No) |  |  |  |  |
| Yes | 5.3 | 10.3 | 8.8 | 15.1 |
| Alcohol consumption (ref.= No) |  |  |  |  |
| Yes | 22.4 | 14.0 | 15.8 | 42.6 |
| School attendance (ref.= Adequate) |  |  |  |  |
| Inadequate | 29.3 | 27.0 | 22.7 | 27.3 |
